# Cortical structural and functional coupling during development and implications for attention deficit hyperactivity disorder

**DOI:** 10.1038/s41398-023-02546-8

**Published:** 2023-07-11

**Authors:** Shania Mereen Soman, Nandita Vijayakumar, Phoebe Thomson, Gareth Ball, Christian Hyde, Timothy J. Silk

**Affiliations:** 1 Centre for Social and Early Emotional Development and School of Psychology, Deakin University, Burwood, VIC 3125 Australia; 2 Child Mind Institute, New York, NY 10022 USA; 3 Department of Paediatrics, University of Melbourne, Parkville, VIC 3010 Australia; 4 Developmental Imaging, Murdoch Children’s Research Institute, Flemington Road, Parkville, VIC 3052 Australia

**Keywords:** Biomarkers, Predictive markers

## Abstract

Functional connectivity is scaffolded by the structural connections of the brain. Disruptions of either structural or functional connectivity can lead to deficits in cognitive functions and increase the risk for neurodevelopmental disorders such as attention deficit hyperactivity disorder (ADHD). To date, very little research has examined the association between structural and functional connectivity in typical development, while no studies have attempted to understand the development of structure-function coupling in children with ADHD. 175 individuals (84 typically developing children and 91 children with ADHD) participated in a longitudinal neuroimaging study with up to three waves. In total, we collected 278 observations between the ages 9 and 14 (139 each in typically developing controls and ADHD). Regional measures of structure-function coupling were calculated at each timepoint using Spearman’s rank correlation and mixed effect models were used to determine group differences and longitudinal changes in coupling over time. In typically developing children, we observed increases in structure-function coupling strength across multiple higher-order cognitive and sensory regions. Overall, weaker coupling was observed in children with ADHD, mainly in the prefrontal cortex, superior temporal gyrus, and inferior parietal cortex. Further, children with ADHD showed an increased rate of coupling strength predominantly in the inferior frontal gyrus, superior parietal cortex, precuneus, mid-cingulate, and visual cortex, compared to no corresponding change over time in typically developing controls. This study provides evidence of the joint maturation of structural and functional brain connections in typical development across late childhood to mid-adolescence, particularly in regions that support cognitive maturation. Findings also suggest that children with ADHD exhibit different patterns of structure-function coupling, suggesting atypical patterns of coordinated white matter and functional connectivity development predominantly in the regions overlapping with the default mode network, salience network, and dorsal attention network during late childhood to mid-adolescence.

## Introduction

Brain development during childhood and adolescence is a highly dynamic process characterized by rapid changes in structural and functional connections within and between different regions of the brain [1]. In this context, structural connectivity refers to properties of anatomical connections or white matter tracts connecting different brain regions [2], while functional connectivity is the degree to which spontaneous fluctuations in activity correlate between regions over time [3]. Each form of connectivity supports the efficient processing and integration of information between various regions of the brain [4, 5]. Structural connections are also crucial to facilitate a coordinated neural activity or functional communication [2, 6–11], with the strength of associations between structural and functional brain connectivity termed structure-function coupling [6, 12]. Disruption in functional and structural connectivity can lead to deficits in various cognitive functions and increase the risk for neurodevelopmental disorders such as attention deficit hyperactivity disorder (ADHD) [12]. Thus, further understanding of structure-function coupling during brain development may help us better understand how the joint maturation of white matter connections and functional communication supports typical neuro-cognitive development, as well as the neural correlates of neurodevelopmental disorders like ADHD.

Cross-sectional studies suggest that the degree of structure-function coupling is inversely proportional to the complexity of the function each brain region serves. For instance, lower structure-function coupling has been reported in brain regions responsible for higher-order executive functions and self-control like the frontal and limbic systems, whereas higher structure-function coupling has been reported in regions involved in lower-order sensory processing like the visual system [6]. Cross-sectionally, age-related changes in functional and structural connectivity are similar within brain networks [13, 14], suggesting that the maturation of structural connections supports functional communication within specific functional networks (e.g., default mode network (DMN)) [14]. Moreover, structure-function coupling studies in adults have observed strong relationships between structural and functional connections in brain networks connecting frontal, parietal, and cerebellar regions [15–18]. However, little is known about how structure-function coupling, which is important for the development of complex cognitive functions, changes across childhood to adolescence.

Two cross-sectional studies examined associations between age and structure-function coupling during childhood and did not identify any age-related effects in the association between structural and functional connections in default mode, frontoparietal, or salience networks [19, 20]. The only longitudinal study to examine developmental changes in structure-function coupling reported that frontal regions, involved in complex higher-order executive functions, showed increased structure-function coupling throughout childhood and adolescence [6]. Moreover, it has been reported that higher structure-function coupling in the lateral prefrontal cortex is associated with improved executive functions [6]. Together, these results suggest that it is important to examine the development of structure-function coupling across the whole brain to understand how various circuits specialize over time to support the emergence of cognitive processes [6]. An examination of developmental changes in structure-function coupling may also shed light on the neural underpinnings of developmental disorders in which individuals exhibit impairments in cognitive processes and functioning, such as ADHD.

ADHD is a prevalent neurodevelopmental disorder that has been associated with aberrant structural and functional network organization during development, predominantly within higher-order cognitive and sensory regions [21–27]. Examining the association between structural and functional connectivity will provide an extensive understanding of the networks disrupted in the pathophysiology of the disorder, which is important for various cognitive functions [28]. To date, however, only two cross-sectional studies have investigated structure-function coupling in children with ADHD. Lee and colleagues observed higher structure-function coupling in children with ADHD compared to typically developing children (5–17 years) within the frontoparietal network (FPN) and DMN [28]. Another study by Bos and colleagues used a data-driven whole-brain approach to investigate both structural and functional connectivity in young children and adolescents with and without ADHD (6–16 years) [29]. They found that greater functional connectivity in prefrontal regions in children with ADHD was not accompanied by differences in the underlying white matter structure compared to typical controls. Both studies emphasize the need for longitudinal studies to improve our knowledge of neural development in children with ADHD.

The goal of the current study was to investigate the development of structure-function coupling between late childhood and mid-adolescence, in a longitudinal sample of typically developing children and those with ADHD. As there has only been one longitudinal study that has examined the development of structure-function coupling in typically developing children, the first aim of this study was to examine the longitudinal changes in structure-function coupling in typically developing children. The second aim was to be the first to compare longitudinal changes in structure-function coupling in children with ADHD to typically developing children. We hypothesized that structure-function coupling would increase with age in higher-order cognitive regions in typically developing children. We also hypothesized that there would be significant differences between children with ADHD and typically developing children in structure-function coupling in higher-order cognitive and sensory regions, including differential developmental trajectories in these regions.

## Methods and materials

### Participants

This study used data from a community-based sample of 175 children (91 children with ADHD and 84 non-ADHD controls) between the ages of 9 and 14. All participants were a part of the longitudinal neuroimaging cohort, Neuroimaging of the Children’s Attention Project (NICAP) [30], in Melbourne, Australia. Subjects underwent up to three waves of repeated MRI scans at ~18-month intervals. Screening for ADHD was undertaken using parent and teacher reports on Conners 3 ADHD Index [31], followed by diagnostic confirmation using face-to-face diagnostic interviews with parents (NIMH Diagnostic Interview Schedule for Children IV [DISC-IV] [32]). Further information regarding participants and study design is detailed in [33]. Diagnostic confirmation was initially conducted at recruitment (3 years before neuroimaging) and subsequently repeated at the first wave of neuroimaging. Children with a history of ADHD (i.e., met ADHD criteria at either wave) were included in the ADHD group. The control group had to screen negative to parent and teacher Conners 3 ADHD Index, and not meet the criteria for ADHD in diagnostic interviews.

To ensure the quality of imaging data, functional scans with excessive head motion (mean frame-wise displacement greater than 0.5 mm [34], *n* = 10), scans missing field maps (*n* = 25) and poor-quality DWI scans (hyperintense cerebellum, omission of white matter, problematic bias correction, problem with Freesurfer mask, the presence of excess non-brain voxels *n* = 45) were excluded from the final analysis. No significant differences were observed between included and excluded participants in the age distribution of control or ADHD groups in one, two, or three waves (range *p* = 0.062–0.960). However, those children with ADHD who were excluded had more severe ADHD symptoms than included ADHD subjects (*p* < 0.05).

**Fig. 1:**
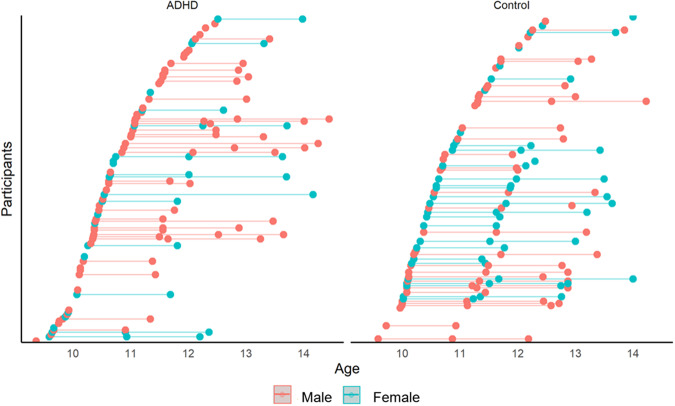
The distribution of participants with ADHD and those without ADHD. Distribution of ADHD and control participants who met inclusion criteria for both resting-state fMRI and DWI data.

The final sample with both structural and functional data comprised 278 scans (139 Control, 139 ADHD) across the three assessment waves (see Fig. 1 and Table 1). At any given wave 7–21% of the ADHD group were taking medication related to their diagnoses, and of this subset, medications comprised methylphenidate: 90–100%, atomoxetine: 0–10%. In addition to one of the former, 33–50% were concurrently taking clonidine or fluoxetine.

**Table 1:** Demographic characteristics of participants.

	ADHD	Control	Difference
Participants wave 1 (% male)	57 (70%)	48 (56%)	*χ*^2^ = 0.77
Participants wave 2 (% male)	53 (74%)	56 (57%)	*χ*^2^ = 0.08
Participants wave 3 (% male)	27 (62%)	34 (56%)	*χ*^2^ = 0.80
Age wave 1, mean (SD)	10.42 (0.51)	10.41 (0.42)	*t* = 0.71
Age wave 2, mean (SD)	11.83 (0.62)	11.70 (0.44)	*t* = 1.16
Age wave 3, mean (SD)	13.23 (0.74)	13.08 (0.51)	*t* = 0.73
dMRI mean head motion wave 1, mean (SD)	0.44 (0.26)	0.36 (0.11)	*t* = 1.98
dMRI mean head motion wave 2, mean (SD)	0.39 (0.14)	0.38 (0.22)	*t* = −0.03
dMRI mean head motion wave 3, mean (SD)	0.33 (0.12)	0.29 (0.06)	*t* = 1.48
rs-fMRI mean head motion wave 1, mean (SD)	0.19 (0.16)	0.14 (0.16)	*t* = 1.12
rs-fMRI mean head motion wave 2, mean (SD)	0.15 (0.11)	0.14 (0.10)	*t* = 0.45
rs-fMRI mean head motion wave 3, mean (SD)	0.11 (0.07)	0.09 (0.05)	*t* = 1.02
DSM inattentive symptom count, mean (SD)	5.59 (2.49)	1.02 (1.42)	*t* = −21.80*
DSM hyperactive-impulsive symptom count, mean (SD)	6.71 (1.69)	0.62 (0.95)	*t* = −10.60*
Baseline symptom severity count (Conner 3 ADHD index), mean (SD)	13.17 (4.69)	1.12 (1.97)	*t* = −16.38*
Medicated wave 1 (%)	12 (21%)	-	-
Medicated wave 2 (%)	10 (19%)	-	-
Medicated wave 3 (%)	2 (7%)	-	-

*p < 0.0001, *dMRI* diffusion MRI, *rs-fMRI* resting-state functional MRI.

### MRI acquisition

All participants underwent a 30 min mock (practice) scanner session to get familiarized with the MRI environment. Subsequently, MRI scans were acquired using a 3-Tesla Siemens scanner at a single site. However, waves 1 and 2 were collected on a TIM Trio scanner, and wave 3 was collected after an upgrade to a MAGNETOM Prisma scanner (note that scanner upgrade was accounted for within statistical modeling). Using a 32-channel head coil, functional images were acquired using multi-band accelerated EPI sequences (MB3), with the following parameters: repetition time (TR) = 1500 ms, echo time (TE) = 33 ms, field of view (FOV) = 255 × 255 mm, flip angle (FA) = 85 deg, 60 axial slices, matrix size = 104 × 104, voxel size = 2.5 mm^3^, and 250 volumes acquired covering the whole brain in a 6 min 33 s sequence. Participants were instructed to keep their eyes open and look at a fixation cross. High Angular Resolution Diffusion Imaging (HARDI) data were acquired using a multi-band factor of three with the following parameters: *b* = 2800 sec/mm^2^, 63 slices, matrix size = 110 × 100, voxel size = 2.4 mm^3^, FOV=260 × 260 mm, bandwidth = 1748 Hz, acquisition time = 3 min 57 s. T1 weighted images were acquired using a multi-echo magnetization prepared rapid gradient-echo (MEMPRAGE) sequence along with navigator-based prospective motion correction with parameters: TR = 2530 ms, TE = 1.77, 3.51, 5.32, and 7.20 ms, FOV = 230 × 230 mm, FA = 7 deg, axial slices = 176, matrix size = 256 × 232, voxel size = 0.9 mm^3^, acquisition time = 6 min 52 s [13].

### Pre-processing of functional data

Pre-processing of resting-state fMRI images was done using FSL 5.0.9 (http://fsl.fmrib.ox.ac.uk/fsl/fslwiki). Standard pre-processing steps such as discarding of four initial volumes to account for initial signal inhomogeneity, motion correction using MCFLIRT (FMRIB’s Linear Registration Tool), B0 unwarping, spatial smoothing using 5 mm FWHM, spatial normalization to the MNI template using a 12-parameter affine transformation and registration of fMRI images to MNI space via high-resolution T1 images using FSL FLIRT and FNIRT were undertaken [35–37]. Further, each preprocessed dataset was decomposed using Multivariate Exploratory Linear Decomposition into Independent Components (MELODIC) in FSL. High-pass temporal filtering (cutoff = 100 s) was also applied to resting-state fMRI data. Following MELODIC, the resulting components from 20 subjects were manually classified as signal or noise based on the previously mentioned criteria [38, 39]. FIX (FMRIB’s ICA-based Xnoisefier) [40] classifier was trained using these classifications. FIX was then run on all single-session MELODIC output to auto-classify Independent Component Analysis (ICA) components into good vs bad components and denoise the data [35]. More details about the denoising method selected for the present study can be found in the Supplementary Material.

### Pre-processing of structural data

Diffusion-weighted imaging (DWI) data was pre-processed using MRtrix3tissue (https://3tissue.github.io), a fork of the MRtrix software [41]. Various commands in MRtrix that work with the help of external software programs such as FSL [42] and ANTS [43] were used to pre-process the raw diffusion images. Pre-processing steps such as denoising [44], Gibbs unringing [45], correction for eddy current, motion [46], bias field [46], and brain mask estimation were performed on all the subjects. Mean frame-wise displacement [47] calculated in each subject’s diffusion space was used for further analysis to reduce motion confounds in diffusion images. After pre-processing the structural data, response functions [48] for white matter, gray matter, cerebero-spinal fluid, and the orientation of fibers in each voxel was estimated (Fiber Orientation Distribution [FOD]) [49]. Further, global intensity differences among the data were corrected using intensity normalization.

### Functional and structural connectome

For each subject, at each wave, functional and structural connectivity matrices were defined using the multi-modal parcellation of human cerebral cortex (HCP-MMP) atlas (360 distinct regions) [50]. The volumetric version of the HCP-MMP atlas available in AFNI [51] was used for the analysis, with the atlas converted and mapped into each subject’s surface space using Freesurfer [52–54]. For the functional connectivity (FC) matrix, Pearson correlation coefficient between each pair of ROIs was calculated using CONN (Functional Connectivity toolbox, CONN20b), resulting in a connectivity matrix of size 360 × 360. Structural connectivity (SC) matrix for each subject at each wave was created by following the steps for estimating the whole brain tractogram outlined in Basic and Advanced Tractography (BATMAN) [55]. Streamlines were created using anatomically constrained tractography [56], and spherical-deconvolution informed filtering of tracks (SIFT) [57]. Further, the SC for each subject at each wave was created using the HCP-MMP atlas by scaling contribution of each streamline to the connectome edge by the inverse of the two node volumes [50]. The options “-symmetric” and “zero_diagonal” in MRtrix were used for generating symmetric SC with diagonals set to zero. Further, structural connectivity matrices were thresholded using consistency-based thresholding at the 75th percentile for edge weight coefficient of variation to reduce the influence of false positives and false negatives, and nodes with just zero values were excluded, as suggested in prior research [6]. The code used for consistency-based thresholding is publicly available on github: https://github.com/shaniasoman/SC_consistency-based_thresholding/blob/main/SC_thr.m.

### Structure-function coupling

**Fig. 2:**
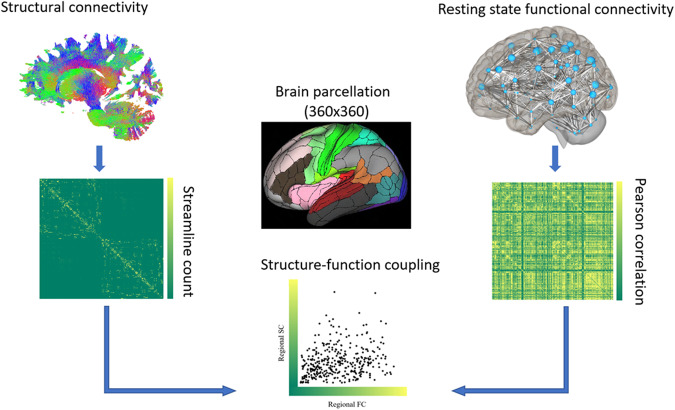
Illustration describing how structure-function coupling is measured using resting-state functional and structural networks. Nodes for resting-state functional and structural networks were defined using the multi-modal parcellation of human cerebral cortex atlas (360 × 360) [6]. Structure-function coupling for each region was calculated by performing Spearman’s rank correlation between the non-zero elements of structural and functional connectivity of each region to the average of every other region of the brain.

The structural and functional connectome of 278 datasets (139 Control, 139 ADHD) were used to examine structure-function coupling between late childhood and mid-adolescence (Fig. 2). To calculate structure-function coupling for each region, Spearman’s rank correlation was performed between the non-zero elements of structural and functional connectivity of each region to the average of every other region of the brain [6]. The code for structure-function coupling is publicly available on github: https://github.com/shaniasoman/SC_FC_coupling/blob/main/SC_FC_coupling.R.

### Developmental trajectories of structure-function coupling

Developmental changes of structure-function coupling were examined using generalized additive mixed models (GAMM), to account for longitudinal data and the possibility of linear and nonlinear trajectories. All models included mean frame-wise displacement of structural and functional connectivity of each subject, scanner effect (pre vs post upgrade), sex, and medication as covariates. GAMMs were implemented in R 4.0.3, with the package ‘mgcv’ [58].

First, we examined age-related changes in structure-function coupling in typically developing children. We compared (i) a null model to (ii) a main effect of age, in predicting structure-function coupling. Next, we included children with ADHD. To estimate the differential developmental trajectories in children with ADHD relative to typically developing children four different models were used: (i) a null model, (ii) main effect of age (iii) main effect of group, and (iv) the interaction of group and age. For all models, the basic dimension for the smooth term was set to 4 (maximum degrees of freedom for smooth term) as recommended by Wood [59]. Each model was examined using maximum likelihood function. Models were compared with likelihood ratio tests (LRT) and Akaike Information Criterion (AIC) to identify the best-fitting model. More complex models were chosen over lower models based on significant LRT (*p* < 0.05) and AIC units <2 [60]. Further, false discovery rate (FDR) (*p* < 0.05) was used to test the statistical significance of coefficients. All the whole brain maps and trajectory plots were created using Pysurfer v0.10.10 (https://pysurfer.github.io/) and RStudio [58] respectively.

## Results

### Structure-function coupling in typically developing children

**Fig. 3:**
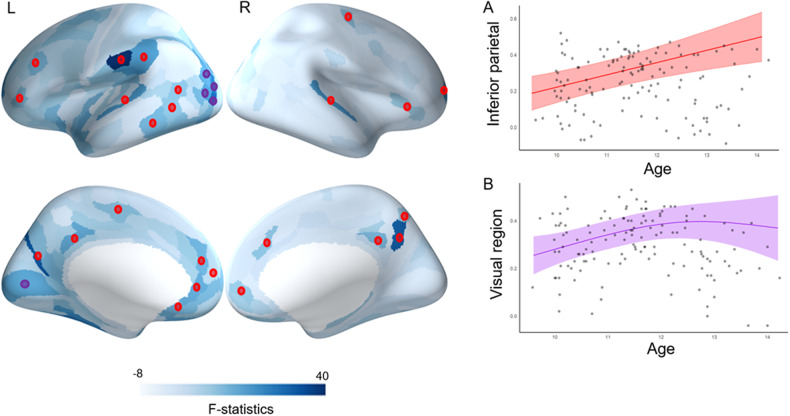
Developmental trajectories of structure-function coupling in typical developing children (main effect of age). **A** Illustration of trajectories shown by higher-order cognitive regions. **B** Illustration of trajectories shown by sensory and visual regions. Red * indicates the higher-order cognitive regions and purple * indicates the sensory and visual regions that survived FDR correction (*p* < 0.05).

First, we evaluated how structure-function coupling develops in typically developing children between the ages of 9 and 14. Age-related differences in structure-function coupling were distributed across the cortex, including the prefrontal, anterior cingulate, mid cingulate, posterior cingulate, precuneus, inferior parietal, middle temporal, and visual cortex (Fig. 3, Figure S6 and Table S2). Notably, regions involved in higher-order cognitive regions showed increased coupling from 9 to 14 years of age, while those involved in sensory and visual processes showed increasing coupling from 9 to 12 years of age followed by a plateau.

### Structure-function coupling in children with ADHD

**Fig. 4:**
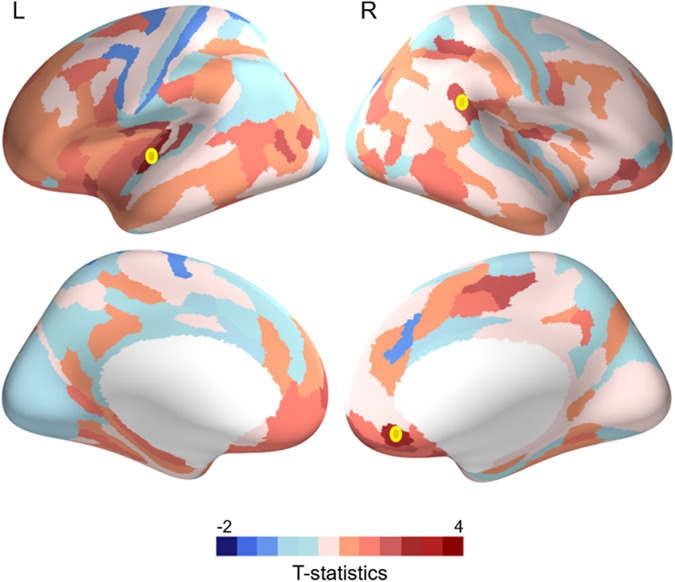
Group differences between ADHD and controls in structure-function coupling. Blue indicates stronger connectivity in ADHD and red indicates stronger connectivity in controls. * Indicates regions that survived FDR correction (*p* < 0.05).

**Fig. 5:**
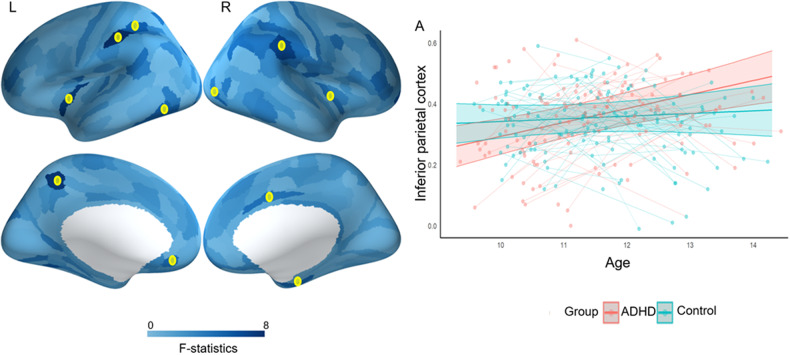
Group differences in developmental trajectories of structure-function coupling (i.e., group x age interaction). * Indicates regions that survived FDR correction (*p* < 0.05). For the regions marked with red *, the ADHD group showed an increase in structure-function coupling with age whereas the control group showed no change from 9 to 14 years of age (as illustrated in **A**).

Next, we examined differences in structure-function coupling in children with ADHD compared to typically developing controls. Across 9–14 years, children with ADHD showed weaker structure-function coupling in the left superior temporal gyrus, right inferior parietal cortex, and right medial prefrontal cortex (Fig. 4 and Table S3). They also exhibited differential trajectories in coupling between 9 and 14 years relative to controls. Children with ADHD showed a significant increase in structure-function coupling in the bilateral inferior frontal gyrus, left medial prefrontal cortex, left superior parietal cortex, left precuneus, left inferior temporal cortex, right inferior parietal, right mid cingulate, right medial temporal cortex, and right visual region, while typically developing children did not exhibit any changes in coupling within these regions (Fig. 5, Figure S7 and Table S4).

## Discussion

The present study examined longitudinal changes in structure-function coupling with typical development and highlighted developmental differences in children with ADHD. We observed a significant change in structure-function coupling in typically developing individuals between late childhood and mid-adolescence in various regions implicated in higher-order cognitive and sensory processes, including the prefrontal, anterior cingulate, mid cingulate, posterior cingulate, precuneus, inferior parietal, middle temporal and visual cortex. We also observed that children with ADHD exhibited differential developmental trajectories to typically developing controls in brain regions such as bilateral inferior frontal gyrus, left medial prefrontal cortex, left superior parietal cortex, left precuneus, left inferior temporal cortex, right inferior parietal, right mid cingulate, right medial temporal cortex, and right visual region.

### Structure-function coupling in typical development

Our longitudinal investigation demonstrated that the typical development of structure-function coupling from late childhood to mid-adolescence was predominantly distributed in brain regions such as prefrontal, anterior cingulate, mid-cingulate, posterior cingulate, precuneus, inferior parietal, middle temporal, and visual cortex. These regions mainly overlap with the areas of DMN, SAL, FPN, sensorimotor, and visual network. Notably, the regions that overlap with higher-order networks (DMN, SAL, FPN) showed increased structure-function coupling from 9 to 14 years of age, while regions that overlap with the sensorimotor networks (SMN and visual networks) showed increased structure-function coupling from 9 to 12 years of age followed by a plateau to 14 years of age. These findings are consistent with the only prior longitudinal study examining developmental trajectories of structure-function coupling from childhood to adulthood, which noted increased coupling of DMN and FPN regions over this time [6].

Studies have observed that protracted changes in coupling are crucial for the development of complex cognitive functions across childhood to adolescence [6, 61]. Several cross-sectional studies have observed strong associations between structural and functional connections [62–64]. However, the association between structural and functional connections is not always straightforward; strong functional interactions can be observed between regions with little or no structural connection (e.g., via a third common connection [9]). Additionally, though strong correlations may be observed between structural and functional connectivity, it can be unclear whether the development of white matter supports functional communication or vice-versa. Our findings do not examine the causal relationship between structural and functional connectivity as their association is measured by correlation. The present evidence suggests that this strong association could be due to changes in functional or structural connectivity during brain development across late childhood to mid-adolescence. Multiple biological processes driving the development of white matter may support the ongoing development of functional communication [60, 61]; likewise it is possible that the use of brain regions together drives structural development to maintain and facilitate future coordinated functioning [9].

Moreover, there could be some other underlying factors such as myelination or axon diameter that cause strong association between structural-functional connections. For example, while we examined fiber count as our measure of structural connectivity, it is possible that the development of myelin on those fibers or change in axon diameter could facilitate improved functional connectivity [65, 66]. Strong structure-function coupling in the highly myelinated sensory regions and weaker structure-function coupling in less myelinated higher-order cognitive regions has been reported in prior cross-sectional studies [6]. Notably, axons of brain regions associated with higher-order cognitive functions myelinate at a slower rate during childhood and continue to myelinate into adulthood [6, 65], which could contribute to changes in interaction between structural and functional connections. Our results of increased structure-function coupling in higher-order cognitive brain regions may reflect a process of maturation and specialization of higher-order brain regions, which become more efficient and effective over time as they establish stronger connections with other brain regions [67, 68]. For example, as the prefrontal cortex matures, it may develop stronger connections with other regions involved in executive functions and self-control, such as the limbic system [68], and exhibit greater levels of structure-function coupling. It is important to note that this developmental trend does not necessarily contradict the cross-sectional findings of lower structure-function coupling in higher-order brain regions. Instead, it may reflect a different aspect of brain organization that is not fully captured by cross-sectional studies. Comparatively, early increases followed by plateau in coupling of sensory and visual networks suggest potentially earlier patterns of maturation, supported by a range of prior studies showing early development of structural and functional connections in the regions associated with visual and sensory functions [69]. Indeed, earlier development of brain connections in these regions is critical to facilitate reflex behaviour and sensory integration at an early age [70, 71]. Collectively, these findings suggest that structure-function coupling from late childhood to early adolescence is characterized by protracted development of networks associated with higher-order cognitive functions that continues throughout early-to-mid adolescence, and earlier maturation of networks associated with sensory and visual functions.

### Structure-function coupling in ADHD

We also observed group differences in structure-function coupling between children with ADHD and their typically developing counterparts. Across late childhood to mid-adolescence, those with ADHD showed reduced coupling in brain regions involved in SAL and DMN (left superior temporal gyrus, right inferior parietal region, and right medial prefrontal cortex) compared to controls. Abnormalities in these regions and networks have been consistently reported in prior cross-sectional structural and functional connectivity studies of ADHD compared to controls [72–81], with delayed myelination thought to contribute to higher symptoms in those with ADHD [82]. Moreover, lower structure-function coupling has previously been related to higher ADHD symptoms in adults [83]. There have only been two cross-sectional studies that have examined group differences in structure-function coupling between typically developing children and children with ADHD [28, 29]. Bos and colleagues failed to identify any significant differences in structure-function coupling [29] (ADHD = 35, controls = 26), which may reflect limited power in the sample size to identify significant effects of the dynamics in structure-function coupling during development. The study has highlighted the importance of longitudinal cohorts to illustrate developmental changes in structure-function coupling. On the other hand, Lee and colleagues observed higher coupling within DMN and FPN in the combined subtype of ADHD than in controls [28]. This contradicts our findings of reduced structure-function coupling in ADHD relative to controls, though may be due to the wide age range (6–17 years, total *N* = 210 (70 children with ADHD-C, 75 children ADHD-I and 56 TDC)) or different metrics (global efficiency) used in their work [29]. Overall, the findings suggest that disruption in structure-function coupling of DMN, and SAL could contribute to at least some of the dysfunctions observed in children with ADHD. However, future studies exploring the association between the development of structure-function coupling and neurocognitive measures are required to confirm this hypothesis.

Given the profound changes in the brain across childhood to adolescence, it is important to examine how structure-function coupling changes with age. We found differential development of structure-function coupling in children with ADHD compared to controls, predominantly in the regions overlapping with the DMN, SAL, DAN, and visual network (left superior temporal gyrus, right inferior parietal cortex, and right medial prefrontal cortex). In particular, those with ADHD showed increasing structure-function coupling with age in these regions, whereas typically developing children showed no change from late childhood to mid-adolescence. Increased structure-function coupling in ADHD for age and group interaction could reflect changes in the neural mechanisms underlying ADHD over time [29]. As individuals with ADHD grow older, they may develop compensatory mechanisms that help them overcome some of the deficits associated with the disorder [84, 85]. These compensatory mechanisms could involve changes in brain structure and function [86] that result in a higher level of structure-function coupling in individuals with ADHD relative to typically developing individuals at certain ages as observed in the current study. This finding emphasizes the dynamic characteristics of ADHD and highlights the significance of employing a longitudinal approach to investigate developmental changes in ADHD. Children with ADHD have been reported to have aberrant structural connectivity predominantly in fronto-striatal connections and other tracts connecting parietal, temporal, and left occipital regions [87, 88]. Moreover, disruptions in functional connections have been demonstrated in the FPN, DMN, DAN, and visual network, and prior cross-sectional studies [76, 80, 89–91], and our recent longitudinal study has reported age-related differences in functional connections between various higher-order cognitive networks [35]. Previous studies demonstrating aberrant functional and structural connections in DMN, SAL, DAN, and visual network have suggested that this could be the reason for deficits in attention, impulsivity, and executive functions in children with ADHD [79, 92–94]. The differential developmental trajectories of structure-function coupling observed in children with ADHD could therefore be due to the disrupted development of structural and/or functional connections [95]. Moreover, these differential developmental trajectories in structure-function coupling could be due to other driving factors such as changes in axon diameter or myelination. Indeed, a range of studies has previously reported that dysregulated myelination is associated with disrupted brain maturation and impairment of various cognitive functions in children with ADHD [96, 97]. In addition, higher structure-function coupling is observed as myelin develops, supporting communication between regions [98]. It is possible that higher coupling seen in ADHD in sensory and visual regions reflects a temporal lag whereby controls have peaked, and ADHD continues to show development into later ages. However, it is difficult to make strong inferences about such “lags” based on the age period examined. Nonetheless, altogether our findings suggest that differences in the maturation of structure-function coupling in higher-order cognitive and visual regions may underlie ADHD, and potentially give rise to cognitive and behavioural deficits typically observed in children with ADHD. Changes in structure-function coupling may also relate to changes in cognitive and behavioural profiles of children with and without ADHD across development—a hypothesis that requires direct examination in future research.

### Limitations and future directions

These findings should be considered in light of certain limitations of our study. Firstly, although we accounted for medication status in statistical models, differences in trajectories between medicated and non-medicated individuals with ADHD were not examined due to the small number of individuals taking medication in our sample. Moreover, we did not examine how changes in structure-function coupling are associated with changes in neurocognitive functioning and changes or remission of symptoms. Future studies examining such relations between structure-function coupling and cognitive and clinical measures would help improve our understanding of the implications of these neural trajectories for developmental outcomes.

In conclusion, our results provide a further understanding of the developmental changes in structure-function coupling in typically developing children, as well as aberrations in these neural patterns in those with ADHD. Findings suggest protracted development of structure-function coupling of higher-order regions in typical development, as well as differential developmental trajectories in structure-function coupling in children with ADHD relative to their peers.

## Supplementary information

Supplementary Material
