# Supplementary material for: Cortical structural and functional coupling during development and implications for attention deficit hyperactivity disorder
Source: Transl Psychiatry. 2023 Jul 11;13:252. doi: 10.1038/s41398-023-02546-8 (PMC10336084; doi:10.1038/s41398-023-02546-8)
Supplement: Supplementary file 1 — Supplementary Material [file 41398_2023_2546_MOESM1_ESM.docx]

Syntax of models used in the study:

Gam1 <- gam(Y ~ s(ID, bs=‘re’) + Covariates)

Gam2 <- gam(Y ~ s(Age, k=4) + s(ID,bs=‘re’) + Covariates)

Gam3 <- gam(Y ~ s(Age, k=4) + Group + s(ID,bs=‘re’) + Covariates

Gam4 <- gam(Y ~ s(Age, k=4) + Group + s(Age, by=Group, k=4) + s(ID,bs=‘re’)+ Covariates)

Covariates used in the study – Sex, Medication, Scanner effects

*Denoising approach used in the study:*

We established the best denoising approach for our data by conducting a pilot study using three well-known pre-processing pipelines on 10 participants of the NICAP functional data. The pipelines are: 1) Denoising pipeline in CONN toolbox, which involves linear regression of the potential confounding effects in the resting state signal, followed by temporal band-pass filtering 2) ICA-based Automatic Removal of Motion Artifacts (ICA-AROMA) and 3) FMRIB’s ICA-Based Xnoisefier (FSL-FIX). CONN toolbox uses an anatomical component-based noise correction procedure (aCompCor) to remove noise components from white matter and cerebrospinal areas for each voxel. Prior research has shown that denoising using the CompCor approach shows good performance only on low motion data (1-4). On applying denoising approach in the CONN toolbox on NICAP pilot data, we observed inter-subject variability, and an inappropriate centered distribution (see Figure S1). Hence, we tried more efficient approaches, ICA-AROMA and FSL-FIX, in removing the artefact components from the BOLD signal.

*
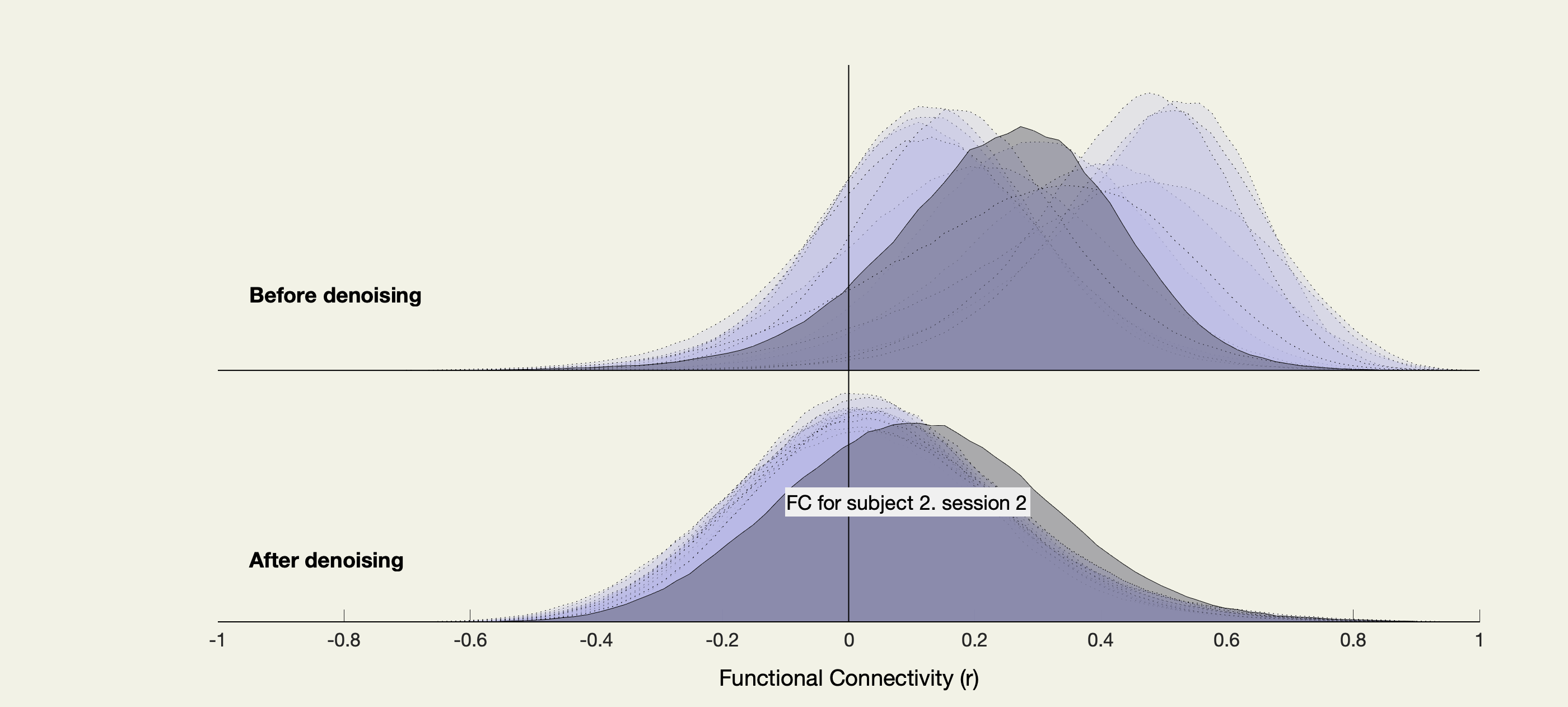
*

*Figure S1. Functional connectivity of the pilot data before and after denoising approach using CONN toolbox*

*ICA-AROMA uses FSL’s MELODIC (5) to decompose the BOLD signal into independent components (IC) and auto-classify the components as signal or noise. The identified noise components are further removed from the BOLD signal using linear regression. For the pilot study, we used ICA-AROMA in FSL to automatically identify and remove noise components from BOLD signal. ICA-AROMA was applied to smoothed resting-state fMRI data, prior to temporal filtering. We explored whether this approach was able to classify “signal” vs “noise” components automatically. However, we found some misclassification in the components i.e. signals were classified as noise (see Figure S2). Of note, this approach does not have an option to create training data using our own participants. This may have biased the results as the training data available in the pipeline did not match the age of our population of interest.*

*
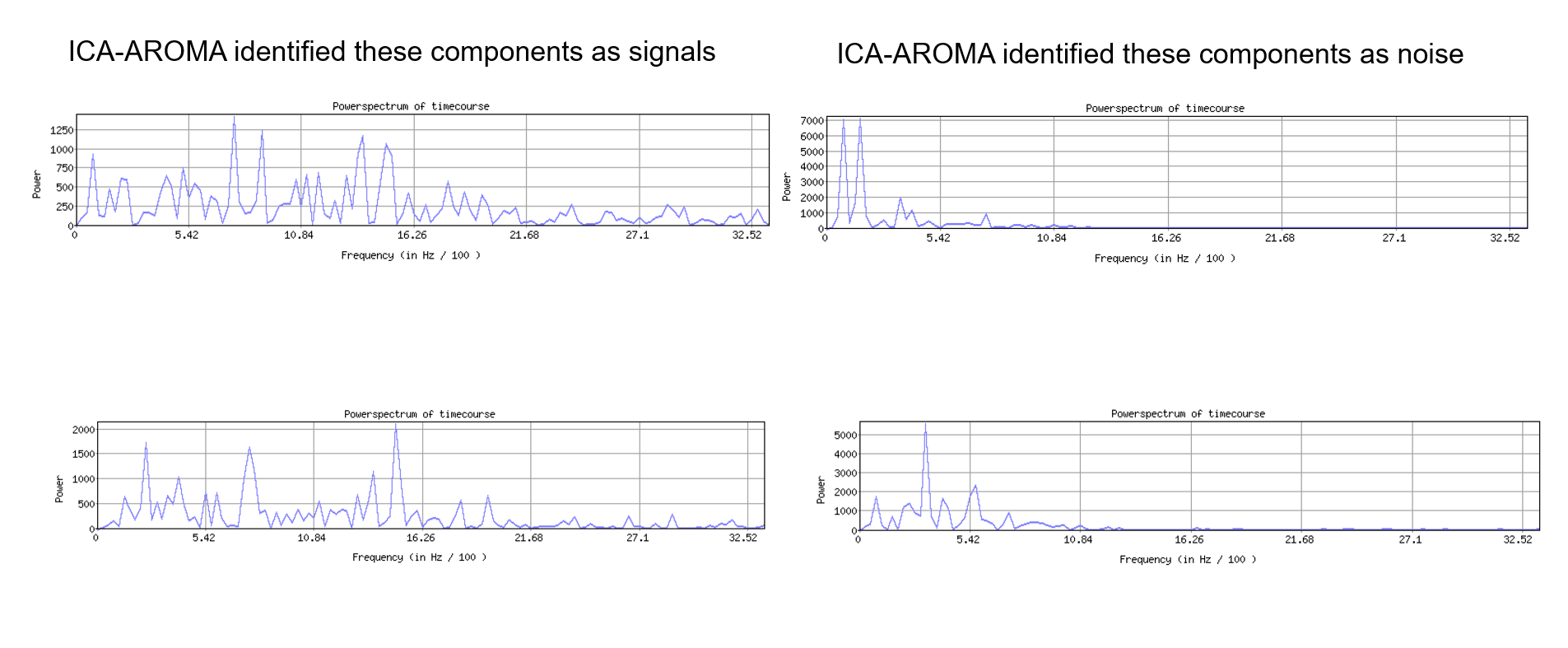
*

*Figure S2. Classification of components by ICA-AROMA*

*Further we used the denoising approach FSL-FIX on our pilot data. FSL-FIX is another ICA-based denoising approach that auto-classifies the components into signal or noise, however it is highly recommended to use the study specific training data. For the pilot study, preprocessed resting-state fMRI of the 10 subjects from NICAP data was decomposed using Multivariate Exploratory Linear Decomposition into Independent Components (MELODIC) in FSL. High pass temporal filtering (cut off = 100s) was also applied to the resting-state fMRI data before ICA when using FIX. Training data consisting of 20 subjects were constructed from NICAP data to perform denoising using FSL-FIX. The resulting components from 20 subjects were manually classified as signal or noise based on the criteria’s described by Griffanti and colleagues (Gholamreza et al., 2014; Griffanti et al., 2017; Griffanti et al., 2014. Components which cannot be exactly classified as signal or noise were categorized as unknown. Each component was manually classified by first examining the thresholded spatial map (absolute threshold at Z = 2.5), then the temporal power spectrum and the time series (see Figure S3). The spectral power of the good components usually lies between 0 to 0.05 Hz. However, each component was cross-checked with its spatial map to make sure that the voxels are not at the edges of the brain, ventricles, arteries, or sagittal sinus. If so, those components were classified as noise even though the spectral power was between 0 to 0.05 Hz. Further to create training dataset, a text file labeled hand_labels_noise.txt with a list of bad components in a single line was created in MELOCIC output directory of each training data (eg: [ 1, 39, 52]). After all the hand labels are created, classifier was trained using the trained weights file (Training.RData). FSL-FIX is then applied on each subject using the trained weights file with a threshold of 20. This threshold was chosen from the results of leave-one out approach in FSL-FIX (Table S1), suggesting that a threshold of 20 for this data results in a mean true positive rate (TPR) of 99.4% and true negative rate (TNR) of 99.4%.The structured noises (motion artefacts, MRI scanner effects, non-neural physiology) are further eliminated by regressing the bad ICA time courses and 24 motion parameters out of the preprocessed BOLD data (6).*

*The FSL-FIX result of each subject was visually inspected to make sure that components are accurately classified as signal and noise. Both ICA-AROMA and FSL-FIX were able to eliminate structured noise. However, trained FSL-FIX with NICAP data identified almost all the signals whereas ICA-AROMA missed some signals. Thus, FSL-FIX with trained weights was deemed to be the best denoising approach for NICAP.*

*Table S1.* Results of leave-one out approach in FSL-FIX

| ***FSL-FIX threshold*** | ***5*** | ***10*** | ***20*** | ***30*** | ***40*** | ***50*** |
| --- | --- | --- | --- | --- | --- | --- |
| *TPR (mean)* | *99.6* | *99.2* | ***99.4*** | *98.4* | *98.2* | *97.3* |
| *TNR (mean)* | *96.3* | *98.2* | ***98.4*** | *98.7* | *98.8* | *98.9* |
| *TPR (median)* | *100* | *100* | ***100*** | *99* | *99* | *98* |
| *TNR (median)* | *98.2* | *98.5* | ***99.3*** | *99.5* | *99.6* | *99.7* |


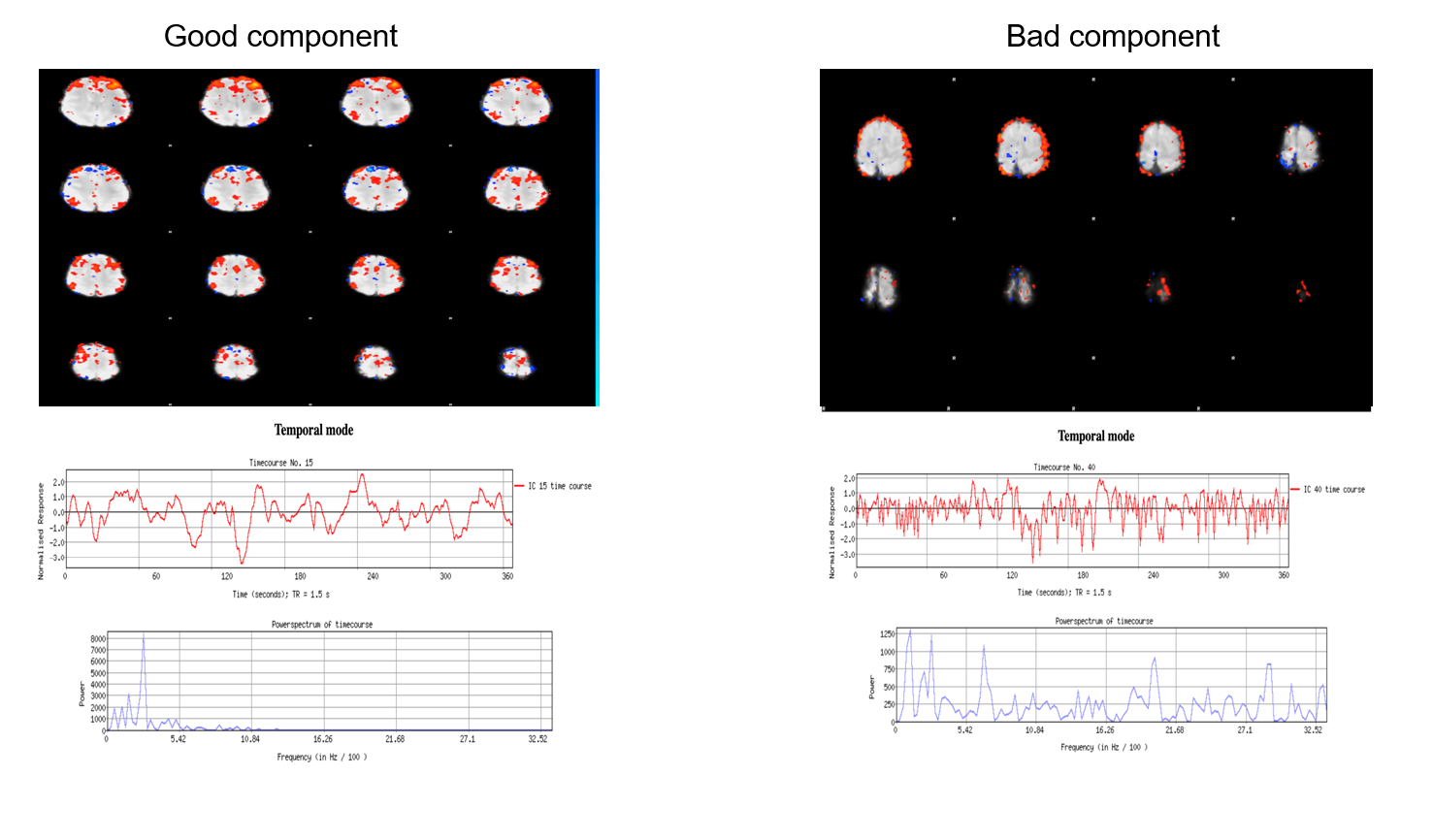


*Figure S3.* Classification of components into good and bad by FSL-FIX

*Further, we have used framewise displacement (FD), a measure that indexes the movement of the head from one volume to the next, of resting-state fMRI data and DWI data in all our models to reduce motion-related artefacts (see Table 1 in the manuscript)*.


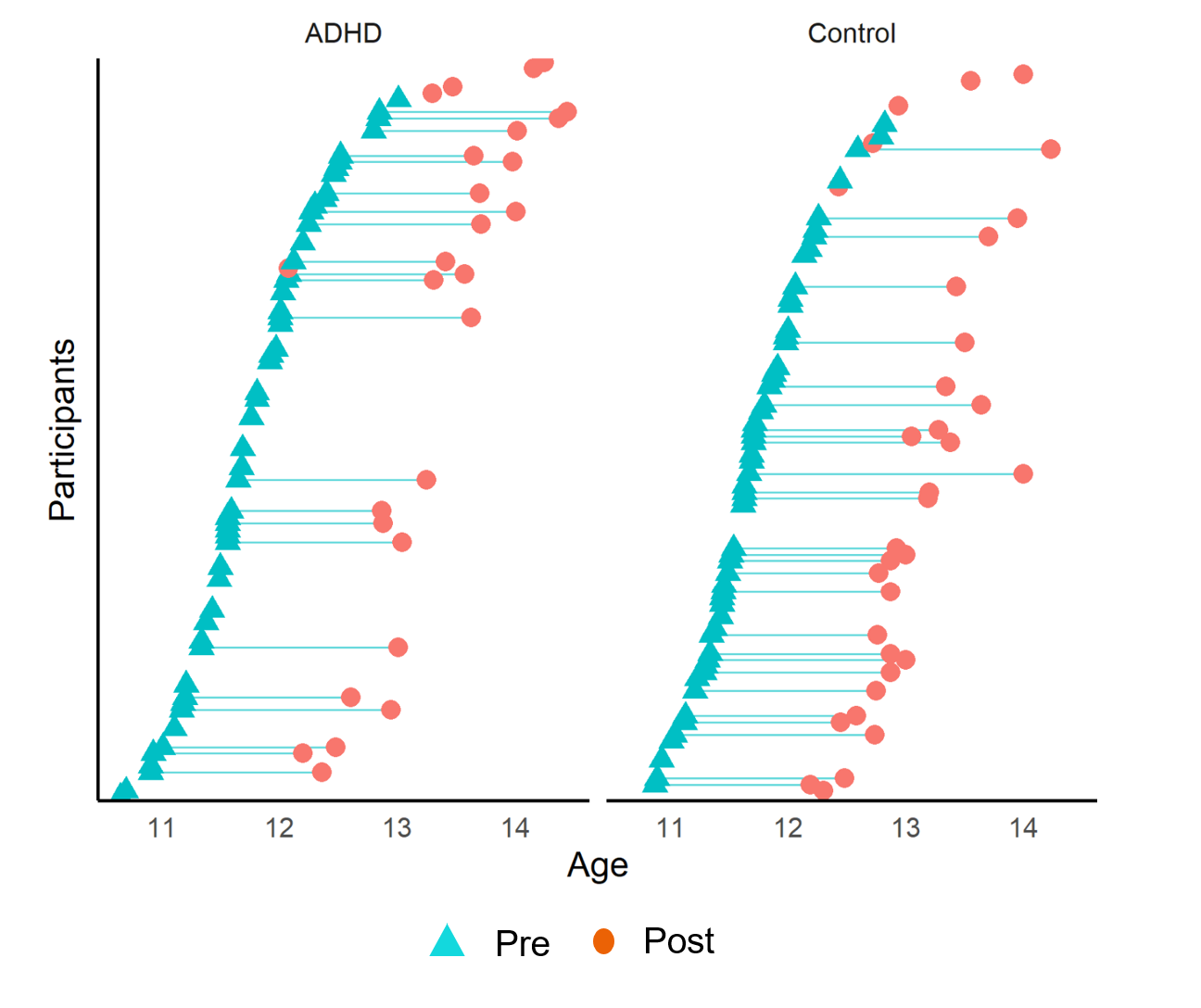


*Figure S4*. Distribution of ADHD and Control participants who completed scans prior and following the scanner upgrade


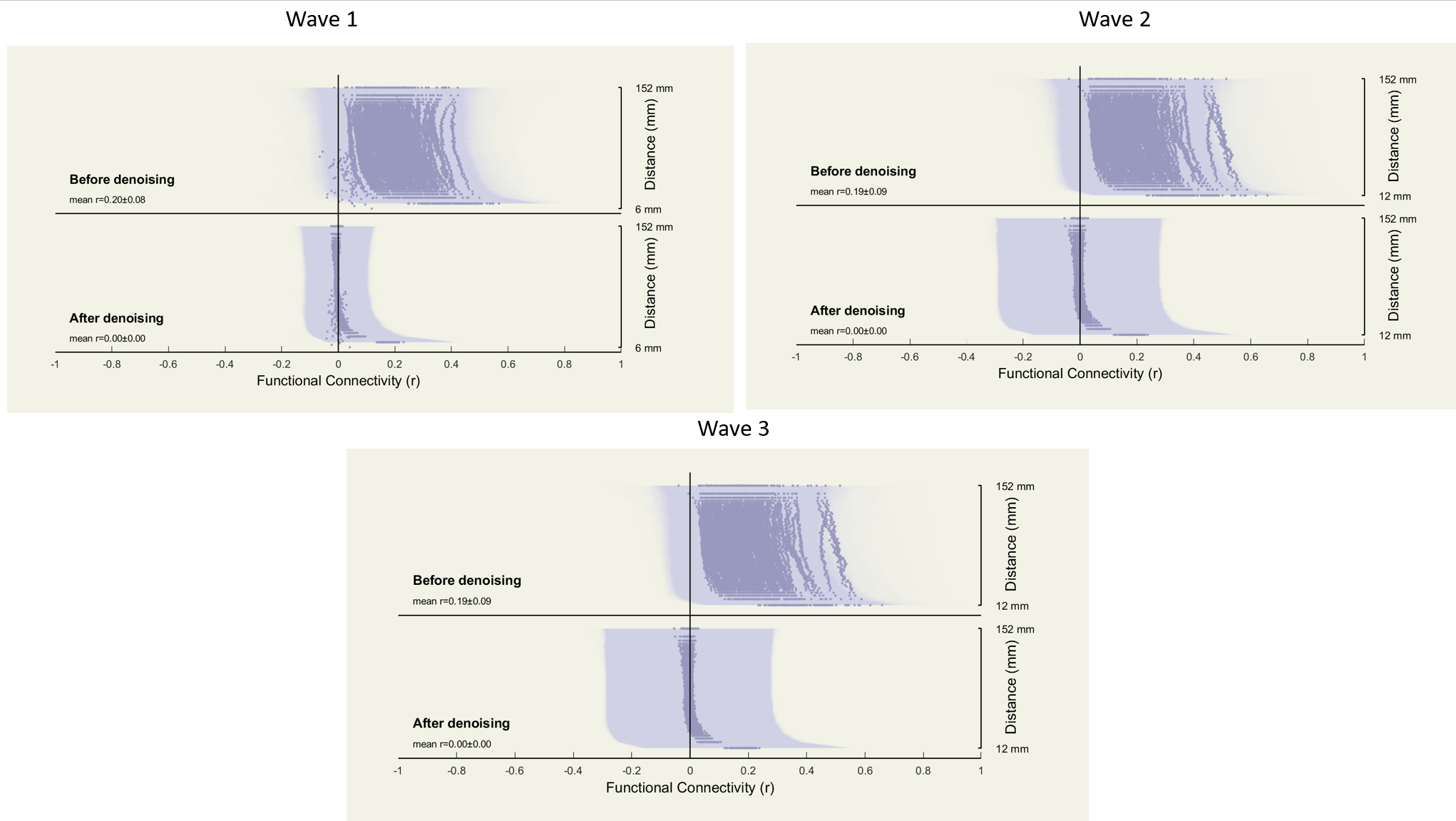


*Figure S5.* Scatterplot of resting-state functional connectivity values (r) against voxel-voxel distance (mm) for wave 1, wave 2 and wave 3 data before and after denoising.


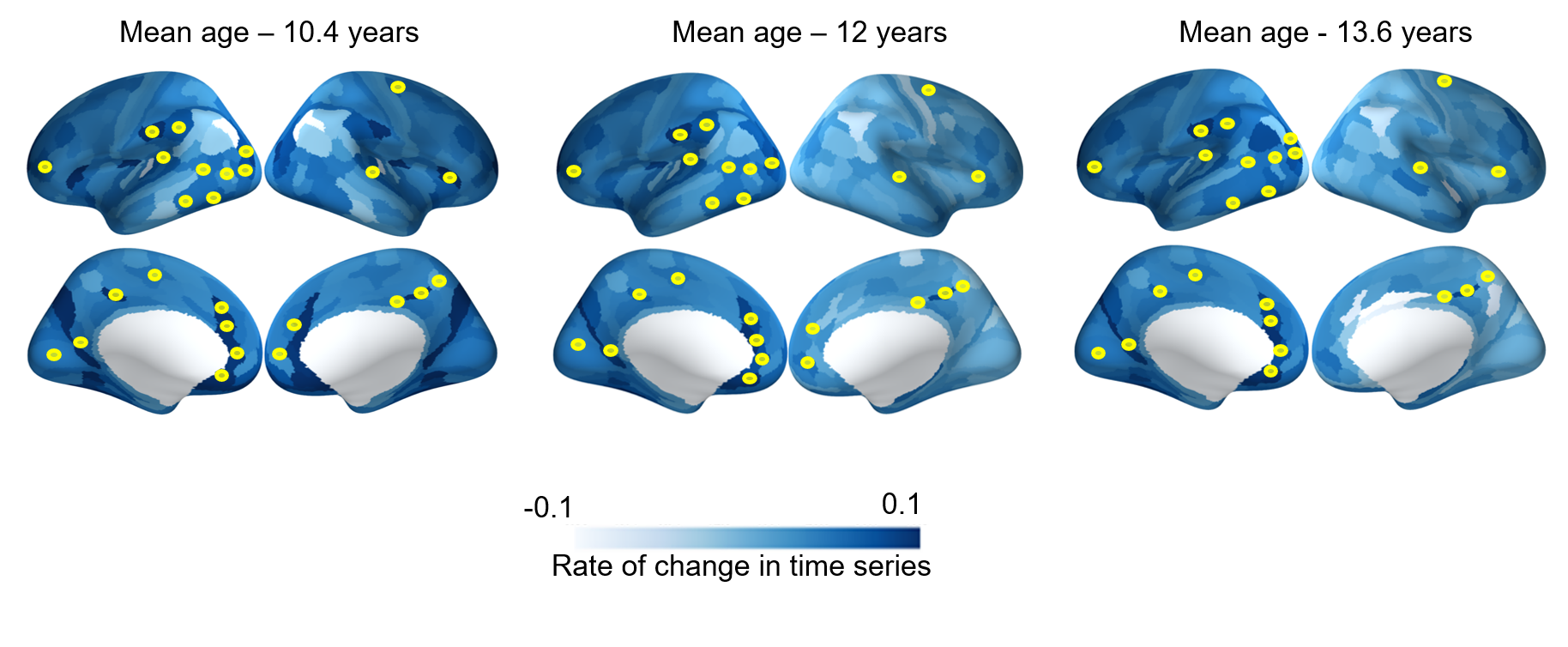


*Figure S6*. Illustration of the developmental change of structure-function coupling in typically developing children across 10 to 14 years of age. Yellow circle indicates age effects that survived FDR correction (*p*<0.05).


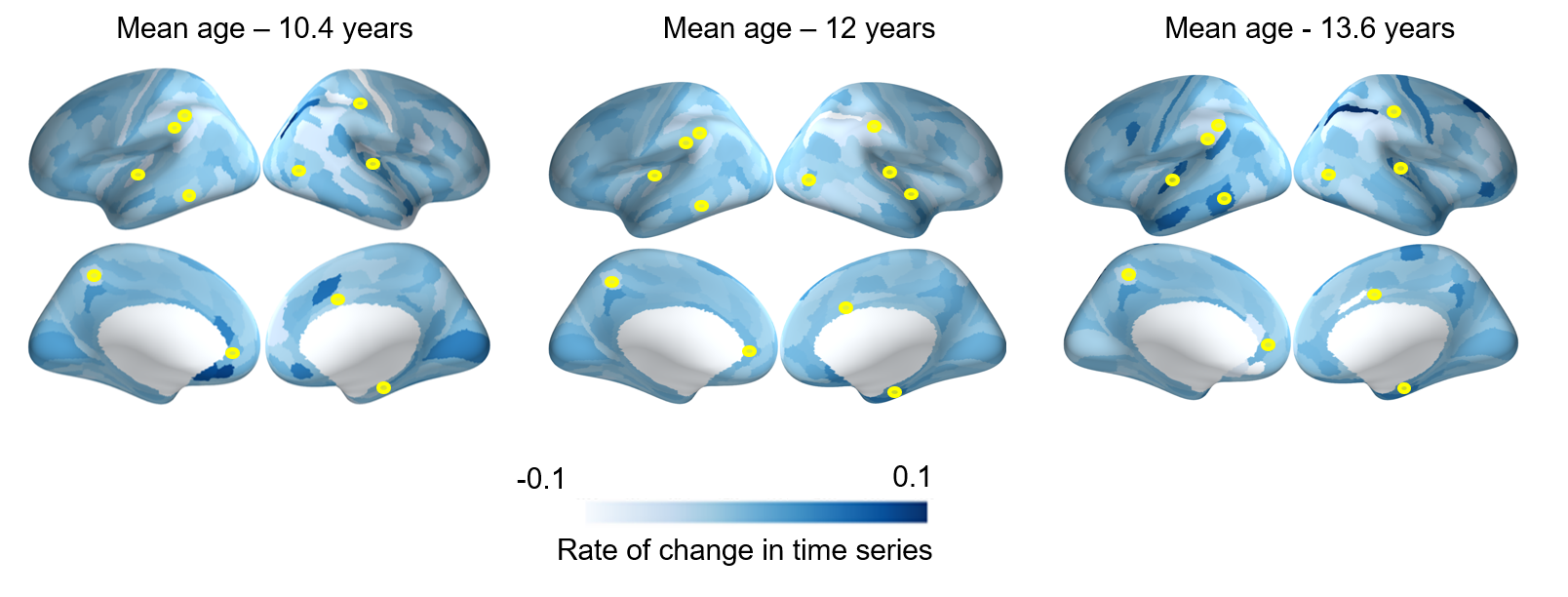


*Figure S7*. Illustration of the differential developmental changes of structure-function coupling in children with ADHD compared to typically developing children across 10 to 14 years of age. Yellow circle indicates group x age effects that survived FDR correction (*p*<0.05).


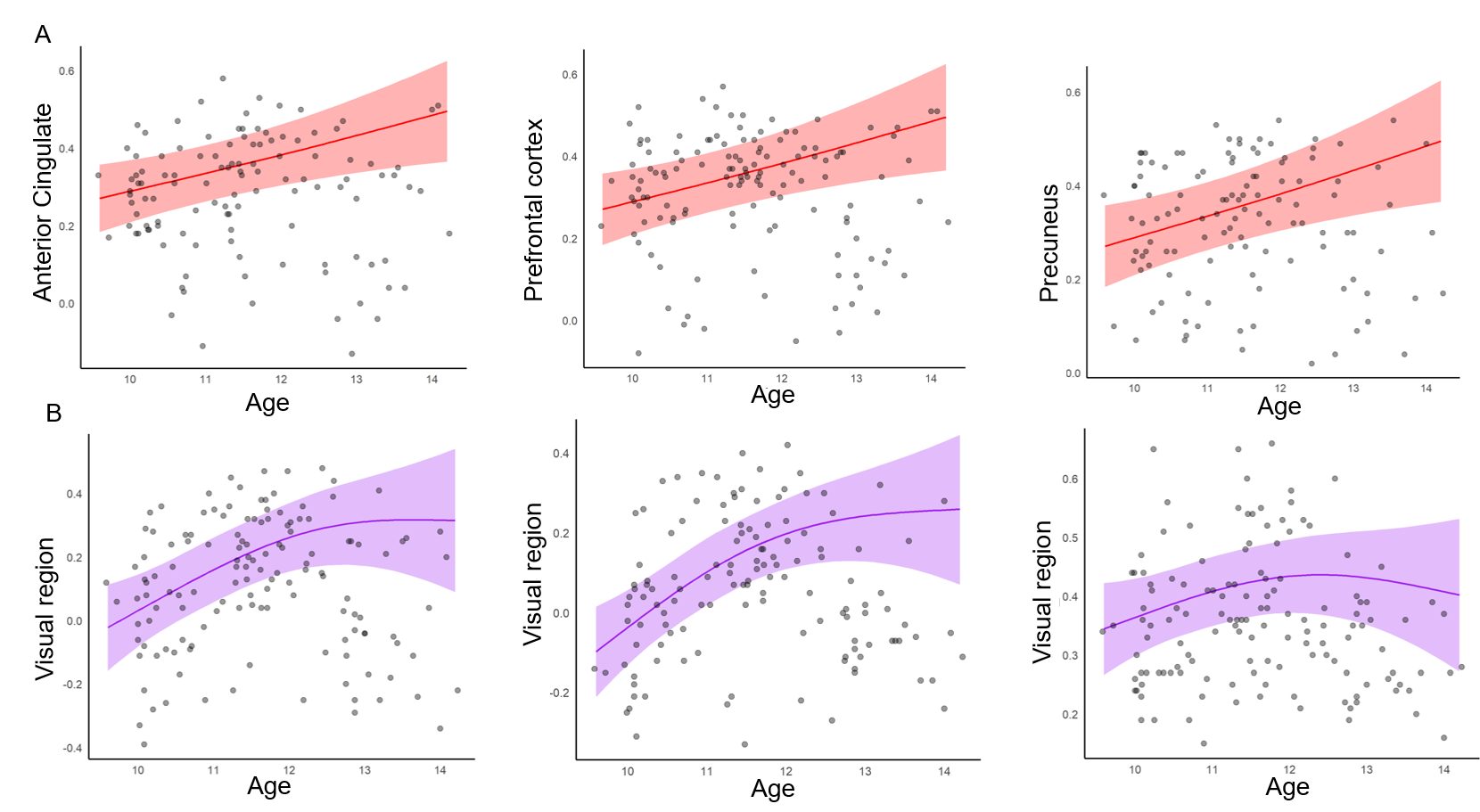


*Figure S8*. Developmental trajectories of structure-function coupling in typical developing children that survived FDR correction (*p*<0.05) (main effect of age). A) Illustration of trajectories shown by higher-order cognitive regions. B) Illustration of trajectories shown by visual regions. All the regions in B are the regions in visual cortex involved in dorsal stream of visual processing.


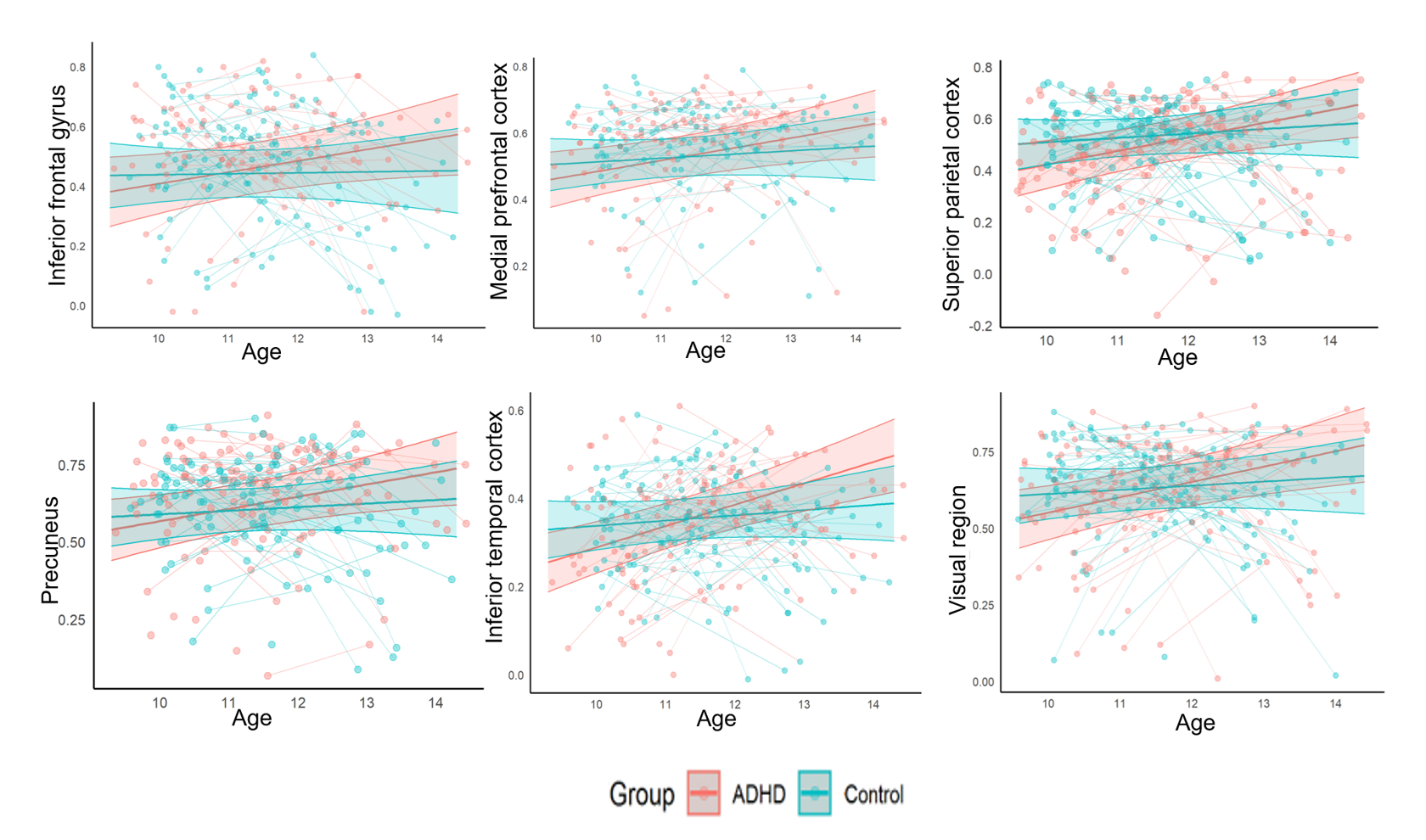


*Figure S9*. Group differences in developmental trajectories of structure-function coupling (i.e., group x age interaction). For all the regions in the plot, the ADHD group showed an increase in structure-function coupling with age whereas the control group showed no change from 9 to 14 years of age

*Table S2.* Statistics for developmental trajectories of structure-function coupling in typically developing children (main effect of age)

| Regions | edf | Ref.df | F | p-value | FDR |
| --- | --- | --- | --- | --- | --- |
| L prefrontal (10d) | 1.00 | 1.00 | 9.40 | <0.001 | 0.005 |
| L anterior cingulate (p24pr) | 1.00 | 1.00 | 11.32 | <0.001 | <0.001 |
| L anterior cingulate (a24pr) | 1.00 | 1.00 | 11.22 | <0.001 | <0.001 |
| L posterior cingulate (POS1) | 1.00 | 1.00 | 10.24 | <0.001 | 0.002 |
| L polar frontal cortex (11L) | 1.00 | 1.00 | 10.26 | 0.004 | 0.030 |
| L mid cingulate (24dd) | 1.00 | 1.00 | 8.35 | <0.001 | 0.008 |
| L inf temporal (TF) | 1.00 | 1.00 | 8.21 | <0.001 | <0.001 |
| L inf temporal (TE2a) | 1.00 | 1.00 | 8.31 | <0.001 | <0.001 |
| L visual cortex (V6) | 2.76 | 2.95 | 17.05 | <0.001 | <0.001 |
| L superior temporal gyrus (A4) | 1.00 | 1.00 | 8.31 | 0.004 | 0.039 |
| L superior parietal cortex (LIPv) | 1.00 | 1.00 | 8.22 | 0.004 | 0.043 |
| L posterior cingulate (31pv) | 1.00 | 1.00 | 8.15 | <0.001 | 0.001 |
| L inferior parietal (PF) | 1.00 | 1.00 | 9.58 | 0.002 | 0.043 |
| L prefrontal cortex (46) | 1.00 | 1.00 | 9.48 | 0.002 | 0.025 |
| L visual area_(LO1) | 2.00 | 2.00 | 30.00 | <0.001 | <0.001 |
| L visual area (LO2) | 2.32 | 2.44 | 14.38 | <0.001 | 0.005 |
| L visual area (LO3) | 2.24 | 2.53 | 20.00 | <0.001 | <0.001 |
| L orbitofrontal cortex (OFC) | 1.00 | 1.00 | 17.42 | <0.001 | 0.001 |
| R medial prefrontal cortex (s32) | 1.00 | 1.00 | 8.31 | 0.004 | 0.039 |
| R inferior frontal cortex (IFJp) | 1.00 | 1.00 | 8.21 | 0.004 | 0.036 |
| R medial prefrontal (p32) | 1.00 | 1.00 | 11.32 | 0.001 | 0.024 |
| R orbitofrontal cortex (OFC) | 1.00 | 1.00 | 10.74 | 0.001 | 0.026 |
| R sup temporal gyrus (47l) | 1.00 | 1.00 | 9.68 | 0.002 | 0.043 |
| R posterior cingulate (31a) | 1.00 | 1.00 | 9.00 | <0.001 | 0.020 |
| R superior parietal (7PL) | 1.00 | 1.00 | 8.25 | 0.004 | 0.042 |
| R precuneus (v23ab) | 1.00 | 1.00 | 9.76 | 0.002 | 0.024 |

NB: L - left hemisphere and R - right hemisphere. All the region names in the bracket represent the label of the regions in HCP-MMP atlas (7)

*Table S3.* Group differences between ADHD and control in structure-function coupling

| Regions | Estimate | Std.error | t-value | p-value | FDR |
| --- | --- | --- | --- | --- | --- |
| L superior temporal gyrus (6v) | -0.038 | 0.019 | -1.99 | 0.001 | 0.039 |
| R inferior parietal cortex (8C) | 0.009 | 0.02 | 2.77 | 0.001 | 0.033 |
| R medial prefrontal cortex (s32) | 0.029 | 0.012 | 2.45 | 0.002 | 0.046 |

NB: L - left hemisphere and R - right hemisphere. All the region names in the bracket represent the label of the regions in HCP-MMP atlas (7)

*Table S4* Statistics for differential developmental trajectories of structure-function coupling

(group x age interaction)

| Regions | edf | Ref.df | F | p-value | FDR |
| --- | --- | --- | --- | --- | --- |
| L inferior frontal gyrus(IFSa) | 1.00 | 1.00 | 9.40 | 0.007 | 0.032 |
| L precuneus (PFt) | 1.00 | 1.00 | 11.32 | 0.004 | 0.019 |
| L superior parietal cortex (7Pm, 7Am) | 1.00 | 1.00 | 8.67 | 0.009 | 0.042 |
| L medial prefrontal cortex (a24pr) | 1.00 | 1.00 | 10.24 | 0.006 | 0.027 |
| L inferior temporal cortex (TF) | 1.00 | 1.00 | 10.26 | 0.001 | 0.034 |
| R inferior frontal gyrus(IFSa) | 1.00 | 1.00 | 9.36 | 0.007 | 0.034 |
| R mid cingulate(24dd) | 1.00 | 1.00 | 8.50 | 0.009 | 0.045 |
| R inferior parietal cortex (Pfm) | 1.00 | 1.00 | 8.35 | 0.001 | 0.042 |
| R medial temporal cortex (PreS) | 1.00 | 1.00 | 10.33 | 0.001 | 0.034 |
| R visual cortex (V6) | 1.00 | 1.00 | 8.21 | 0.008 | 0.043 |

NB: L - left hemisphere and R - right hemisphere. All the region names in the bracket represent the label of the regions in HCP-MMP atlas (7)

**References**

1. Parkes L, Fulcher B, Yücel M, Fornito A (2018): An evaluation of the efficacy, reliability, and sensitivity of motion correction strategies for resting-state functional MRI. *Neuroimage*. 171:415-436.

2. Power JD, Barnes KA, Snyder AZ, Schlaggar BL, Petersen SEJN (2012): Spurious but systematic correlations in functional connectivity MRI networks arise from subject motion. 59:2142-2154.

3. Van Dijk KR, Sabuncu MR, Buckner RL (2012): The influence of head motion on intrinsic functional connectivity MRI. *Neuroimage*. 59:431-438.

4. Satterthwaite TD, Wolf DH, Loughead J, Ruparel K, Elliott MA, Hakonarson H, et al. (2012): Impact of in-scanner head motion on multiple measures of functional connectivity: relevance for studies of neurodevelopment in youth. *NeuroImage*. 60:623-632.

5. Beckmann CF, DeLuca M, Devlin JT, Smith SMJPTotRSBBS (2005): Investigations into resting-state connectivity using independent component analysis. 360:1001-1013.

6. Gholamreza S-K, Gwenaëlle D, Christian FB, Matthew FG, Ludovica G, Stephen MS (2014): Automatic denoising of functional MRI data: Combining independent component analysis and hierarchical fusion of classifiers. *NeuroImage*. 90:449-468.

7. Glasser MF, Coalson TS, Robinson EC, Hacker CD, Harwell J, Yacoub E, et al. (2016): A multi-modal parcellation of human cerebral cortex. *Nature*. 536:171-178.
